# Supplementary material for: Hot spot prediction in protein-protein interactions by an ensemble system
Source: BMC Syst Biol. 2018 Dec 31;12(Suppl 9):132. doi: 10.1186/s12918-018-0665-8 (PMC6311905; doi:10.1186/s12918-018-0665-8)
Supplement: Supplementary file 2 — All relevant MATLAB code. A simple Matlab implement of our predictor. (ZIP 19,742 kb) [file 12918_2018_665_MOESM2_ESM.zip › Additional file 2/Read ASA/ASA website/dbMPIKT/1JTDA.html]

 NetSurfP - Protein Surface Accessibility and Secondary Structure Predictions

  

|  |  |  |
| --- | --- | --- |
|  |  | NetSurfP - Protein Surface Accessibility and Secondary Structure Predictions  Technical University of Denmark |

  


---

```
# For publication of results, please cite:
# A generic method for assignment of reliability scores applied to solvent accessibility predictions.
# Bent Petersen, Thomas Nordahl Petersen, Pernille Andersen, Morten Nielsen and Claus Lundegaard
# BMC Structural Biology 2009, 9:51 doi:10.1186/1472-6807-9-51
#
# Column 1: Class assignment - B for buried or E for Exposed - Threshold: 25% exposure, but not based on RSA
# Column 2: Amino acid
# Column 3: Sequence name
# Column 4: Amino acid number
# Column 5: Relative Surface Accessibility - RSA
# Column 6: Absolute Surface Accessibility
# Column 7: Z-fit score for RSA prediction
# Column 8: Probability for Alpha-Helix
# Column 9: Probability for Beta-strand
# Column 10: Probability for Coil
E H  1JTDA                  1    0.851 154.815   0.363   0.000   0.001   0.999
E P  1JTDA                  2    0.501  71.078  -0.746   0.321   0.003   0.675
E E  1JTDA                  3    0.679 118.586   1.352   0.694   0.003   0.303
B T  1JTDA                  4    0.139  19.265   0.436   0.858   0.002   0.139
E L  1JTDA                  5    0.272  49.748   0.893   0.938   0.007   0.055
E V  1JTDA                  6    0.642  98.706   1.378   0.938   0.007   0.055
E K  1JTDA                  7    0.397  81.704   2.254   0.970   0.001   0.030
B V  1JTDA                  8    0.025   3.858   1.134   0.970   0.001   0.030
E K  1JTDA                  9    0.418  85.941   2.471   0.970   0.001   0.030
E D  1JTDA                 10    0.585  84.371   2.232   0.970   0.001   0.030
B A  1JTDA                 11    0.103  11.351   0.951   0.970   0.001   0.030
B E  1JTDA                 12    0.096  16.824  -0.922   0.923   0.002   0.076
E D  1JTDA                 13    0.715 103.003   1.132   0.858   0.002   0.139
E Q  1JTDA                 14    0.716 127.895   1.028   0.694   0.003   0.303
B L  1JTDA                 15    0.168  30.834   0.397   0.176   0.004   0.820
E G  1JTDA                 16    0.744  58.584   0.481   0.005   0.015   0.979
B A  1JTDA                 17    0.124  13.698  -0.378   0.005   0.045   0.951
E R  1JTDA                 18    0.381  87.226   1.491   0.004   0.616   0.381
B V  1JTDA                 19    0.082  12.619   1.007   0.001   0.959   0.040
B G  1JTDA                 20    0.029   2.274   0.184   0.001   0.959   0.040
B Y  1JTDA                 21    0.025   5.236   1.016   0.000   0.983   0.017
B I  1JTDA                 22    0.042   7.844   0.894   0.000   0.983   0.017
B E  1JTDA                 23    0.019   3.337   1.267   0.000   0.983   0.017
B L  1JTDA                 24    0.065  11.938   1.102   0.000   0.983   0.017
B D  1JTDA                 25    0.085  12.191   0.184   0.001   0.900   0.099
B L  1JTDA                 26    0.163  29.827  -0.168   0.005   0.262   0.733
E N  1JTDA                 27    0.666  97.444   0.312   0.004   0.085   0.910
E S  1JTDA                 28    0.420  49.224  -0.084   0.005   0.015   0.979
E G  1JTDA                 29    0.447  35.179  -1.386   0.018   0.019   0.964
E K  1JTDA                 30    0.444  91.351   0.963   0.021   0.451   0.528
E I  1JTDA                 31    0.302  55.870   1.289   0.011   0.918   0.071
B L  1JTDA                 32    0.112  20.544   1.243   0.011   0.918   0.071
B E  1JTDA                 33    0.203  35.394   0.491   0.011   0.918   0.071
B S  1JTDA                 34    0.177  20.698  -1.045   0.021   0.756   0.223
E F  1JTDA                 35    0.337  67.596   0.562   0.022   0.552   0.426
E R  1JTDA                 36    0.286  65.540   0.522   0.019   0.141   0.840
E P  1JTDA                 37    0.431  61.159  -1.316   0.018   0.047   0.935
E E  1JTDA                 38    0.673 117.521  -0.528   0.018   0.047   0.935
E E  1JTDA                 39    0.357  62.455   0.527   0.019   0.141   0.840
E R  1JTDA                 40    0.396  90.776   0.778   0.022   0.359   0.619
B F  1JTDA                 41    0.117  23.562   1.363   0.021   0.279   0.699
B P  1JTDA                 42    0.191  27.075   0.035   0.056   0.142   0.802
B M  1JTDA                 43    0.076  15.248  -1.323   0.268   0.043   0.689
B M  1JTDA                 44    0.026   5.303  -0.578   0.430   0.016   0.555
B S  1JTDA                 45    0.050   5.825  -1.248   0.717   0.014   0.269
B T  1JTDA                 46    0.049   6.838  -0.782   0.923   0.002   0.076
B F  1JTDA                 47    0.051  10.196   0.334   0.923   0.002   0.076
B K  1JTDA                 48    0.053  10.923   0.539   0.970   0.001   0.030
B V  1JTDA                 49    0.044   6.747   0.216   0.970   0.001   0.030
B L  1JTDA                 50    0.071  13.055   0.361   0.970   0.001   0.030
B L  1JTDA                 51    0.043   7.837   0.079   0.970   0.001   0.030
B C  1JTDA                 52    0.022   3.103   0.772   0.970   0.001   0.030
B G  1JTDA                 53    0.078   6.115   0.261   0.970   0.001   0.030
B A  1JTDA                 54    0.073   8.056   0.889   0.970   0.001   0.030
B V  1JTDA                 55    0.028   4.273   0.902   0.970   0.001   0.030
B L  1JTDA                 56    0.053   9.723   1.009   0.970   0.001   0.030
E S  1JTDA                 57    0.336  39.379   1.398   0.970   0.001   0.030
B R  1JTDA                 58    0.214  49.006   0.600   0.970   0.001   0.030
B V  1JTDA                 59    0.106  16.292   0.234   0.923   0.002   0.076
E D  1JTDA                 60    0.655  94.443   0.816   0.782   0.003   0.216
E A  1JTDA                 61    0.701  77.206   0.800   0.321   0.003   0.675
E G  1JTDA                 62    0.563  44.324  -0.909   0.053   0.005   0.942
E Q  1JTDA                 63    0.719 128.342   0.321   0.018   0.019   0.964
B E  1JTDA                 64    0.137  24.004  -0.078   0.005   0.015   0.979
E Q  1JTDA                 65    0.485  86.639   1.088   0.005   0.015   0.979
B L  1JTDA                 66    0.170  31.200   0.005   0.018   0.047   0.935
E G  1JTDA                 67    0.628  49.392  -0.164   0.018   0.047   0.935
E R  1JTDA                 68    0.407  93.295   0.448   0.005   0.336   0.660
E R  1JTDA                 69    0.451 103.348   1.297   0.002   0.816   0.182
B I  1JTDA                 70    0.066  12.210   0.866   0.001   0.900   0.099
E H  1JTDA                 71    0.487  88.658   1.509   0.001   0.900   0.099
B Y  1JTDA                 72    0.075  16.006   0.950   0.002   0.816   0.182
E S  1JTDA                 73    0.317  37.117   1.198   0.021   0.451   0.528
E Q  1JTDA                 74    0.603 107.678   0.211   0.125   0.227   0.648
E N  1JTDA                 75    0.618  90.504   0.234   0.191   0.086   0.723
B D  1JTDA                 76    0.174  25.030  -1.374   0.191   0.086   0.723
B L  1JTDA                 77    0.268  48.979  -0.380   0.216   0.235   0.548
E V  1JTDA                 78    0.473  72.715  -0.696   0.125   0.227   0.648
E E  1JTDA                 79    0.567  99.125  -0.945   0.118   0.150   0.732
E Y  1JTDA                 80    0.540 115.334  -2.301   0.018   0.088   0.893
B S  1JTDA                 81    0.319  37.410  -2.239   0.018   0.047   0.935
E P  1JTDA                 82    0.312  44.301  -1.928   0.019   0.141   0.840
E V  1JTDA                 83    0.361  55.470  -0.580   0.022   0.359   0.619
B T  1JTDA                 84    0.264  36.575  -0.697   0.021   0.451   0.528
E E  1JTDA                 85    0.396  69.269  -1.565   0.022   0.359   0.619
E K  1JTDA                 86    0.311  63.870  -0.286   0.021   0.279   0.699
E H  1JTDA                 87    0.448  81.437   0.162   0.019   0.141   0.840
E L  1JTDA                 88    0.413  75.547  -0.523   0.018   0.047   0.935
E T  1JTDA                 89    0.696  96.507  -1.571   0.018   0.019   0.964
E D  1JTDA                 90    0.506  72.857  -0.084   0.053   0.043   0.903
E G  1JTDA                 91    0.534  42.034   0.911   0.064   0.216   0.721
B M  1JTDA                 92    0.086  17.209   1.574   0.064   0.216   0.721
B T  1JTDA                 93    0.178  24.702   1.475   0.191   0.086   0.723
B V  1JTDA                 94    0.038   5.871   0.264   0.975   0.003   0.022
E R  1JTDA                 95    0.289  66.181   1.358   0.970   0.001   0.030
B E  1JTDA                 96    0.182  31.743   0.907   0.988   0.000   0.012
B L  1JTDA                 97    0.019   3.534   1.435   0.988   0.000   0.012
B C  1JTDA                 98    0.027   3.735   1.013   0.988   0.000   0.012
B S  1JTDA                 99    0.251  29.429   1.415   0.988   0.000   0.012
B A  1JTDA                100    0.077   8.485   0.052   0.988   0.000   0.012
B A  1JTDA                101    0.023   2.502   0.949   0.970   0.001   0.030
B I  1JTDA                102    0.040   7.492   0.207   0.975   0.003   0.022
B T  1JTDA                103    0.136  18.905   0.747   0.938   0.007   0.055
E M  1JTDA                104    0.251  50.145   0.494   0.802   0.014   0.185
B S  1JTDA                105    0.035   4.114  -1.253   0.339   0.016   0.645
B D  1JTDA                106    0.169  24.411  -1.190   0.176   0.004   0.820
B N  1JTDA                107    0.083  12.122  -1.003   0.600   0.003   0.397
B T  1JTDA                108    0.200  27.782   0.305   0.970   0.001   0.030
B A  1JTDA                109    0.023   2.557   0.768   0.970   0.001   0.030
B A  1JTDA                110    0.019   2.083   1.037   0.970   0.001   0.030
B N  1JTDA                111    0.103  15.108   0.342   0.988   0.000   0.012
B L  1JTDA                112    0.090  16.442   1.341   0.988   0.000   0.012
B L  1JTDA                113    0.023   4.285   1.001   0.988   0.000   0.012
B L  1JTDA                114    0.041   7.434  -0.019   0.970   0.001   0.030
E T  1JTDA                115    0.407  56.409   0.842   0.923   0.002   0.076
E T  1JTDA                116    0.284  39.391   0.697   0.858   0.002   0.139
B I  1JTDA                117    0.094  17.390   0.042   0.502   0.002   0.495
B G  1JTDA                118    0.322  25.334  -1.278   0.053   0.005   0.942
B G  1JTDA                119    0.264  20.793  -0.779   0.053   0.005   0.942
B P  1JTDA                120    0.257  36.412   0.560   0.694   0.003   0.303
E K  1JTDA                121    0.641 131.792   0.513   0.970   0.001   0.030
E E  1JTDA                122    0.381  66.508   1.430   0.970   0.001   0.030
B L  1JTDA                123    0.026   4.742   0.898   0.970   0.001   0.030
B T  1JTDA                124    0.145  20.070   1.289   0.970   0.001   0.030
E A  1JTDA                125    0.536  59.023   1.626   0.988   0.000   0.012
B F  1JTDA                126    0.231  46.301   1.883   0.988   0.000   0.012
B L  1JTDA                127    0.020   3.699   0.954   0.970   0.001   0.030
E H  1JTDA                128    0.472  85.911   1.766   0.970   0.001   0.030
E N  1JTDA                129    0.669  97.883   1.666   0.923   0.002   0.076
B M  1JTDA                130    0.146  29.115   1.386   0.600   0.003   0.397
E G  1JTDA                131    0.400  31.496  -0.647   0.109   0.005   0.886
B D  1JTDA                132    0.091  13.171  -0.225   0.016   0.005   0.979
E H  1JTDA                133    0.583 106.084   0.240   0.018   0.088   0.893
E V  1JTDA                134    0.417  64.047  -0.277   0.019   0.141   0.840
B T  1JTDA                135    0.064   8.821  -0.197   0.021   0.279   0.699
E R  1JTDA                136    0.311  71.105   0.898   0.021   0.451   0.528
B L  1JTDA                137    0.072  13.220  -0.735   0.021   0.451   0.528
E D  1JTDA                138    0.380  54.816   0.114   0.021   0.279   0.699
E R  1JTDA                139    0.347  79.394  -0.545   0.056   0.142   0.802
E W  1JTDA                140    0.320  76.984  -0.966   0.056   0.142   0.802
E E  1JTDA                141    0.342  59.660  -1.245   0.118   0.150   0.732
E P  1JTDA                142    0.324  45.947  -1.825   0.113   0.087   0.800
E E  1JTDA                143    0.336  58.682  -1.162   0.184   0.043   0.773
B L  1JTDA                144    0.196  35.814  -1.414   0.184   0.043   0.773
B N  1JTDA                145    0.208  30.451  -1.074   0.184   0.043   0.773
E E  1JTDA                146    0.577 100.872  -1.591   0.113   0.043   0.844
E A  1JTDA                147    0.344  37.909  -2.286   0.058   0.017   0.925
E I  1JTDA                148    0.361  66.730  -1.509   0.018   0.019   0.964
E P  1JTDA                149    0.391  55.511  -1.620   0.018   0.019   0.964
E N  1JTDA                150    0.577  84.429  -2.389   0.003   0.003   0.994
E D  1JTDA                151    0.406  58.447  -1.092   0.003   0.003   0.994
E E  1JTDA                152    0.437  76.344  -1.543   0.016   0.005   0.979
E R  1JTDA                153    0.452 103.508   0.283   0.016   0.005   0.979
B D  1JTDA                154    0.141  20.318  -1.475   0.018   0.019   0.964
B T  1JTDA                155    0.140  19.349   0.559   0.018   0.088   0.893
B T  1JTDA                156    0.022   3.079   0.169   0.018   0.088   0.893
B M  1JTDA                157    0.043   8.624   0.853   0.181   0.016   0.803
B P  1JTDA                158    0.020   2.852   0.818   0.923   0.002   0.076
B V  1JTDA                159    0.184  28.219   1.028   0.970   0.001   0.030
B A  1JTDA                160    0.053   5.808  -0.800   0.970   0.001   0.030
B M  1JTDA                161    0.018   3.562   1.332   0.970   0.001   0.030
B A  1JTDA                162    0.020   2.215   0.649   0.970   0.001   0.030
E T  1JTDA                163    0.215  29.834   2.013   0.970   0.001   0.030
B T  1JTDA                164    0.028   3.870   0.451   0.970   0.001   0.030
B L  1JTDA                165    0.021   3.863   1.094   0.970   0.001   0.030
B R  1JTDA                166    0.187  42.892   0.904   0.988   0.000   0.012
B K  1JTDA                167    0.170  35.010   1.139   0.970   0.001   0.030
B L  1JTDA                168    0.028   5.072   0.460   0.970   0.001   0.030
B L  1JTDA                169    0.127  23.199   0.448   0.858   0.002   0.139
E T  1JTDA                170    0.501  69.419   0.822   0.600   0.003   0.397
E G  1JTDA                171    0.494  38.846   0.259   0.176   0.004   0.820
E E  1JTDA                172    0.685 119.687   0.148   0.053   0.005   0.942
B L  1JTDA                173    0.211  38.597  -0.817   0.058   0.017   0.925
B L  1JTDA                174    0.086  15.747  -0.327   0.018   0.019   0.964
E T  1JTDA                175    0.438  60.723   1.037   0.018   0.019   0.964
E L  1JTDA                176    0.575 105.374   0.438   0.923   0.002   0.076
E A  1JTDA                177    0.620  68.368   0.560   0.923   0.002   0.076
B S  1JTDA                178    0.110  12.939  -0.728   0.970   0.001   0.030
B R  1JTDA                179    0.111  25.442   0.303   0.970   0.001   0.030
E Q  1JTDA                180    0.541  96.605   1.001   0.988   0.000   0.012
E Q  1JTDA                181    0.275  49.204   1.808   0.970   0.001   0.030
B L  1JTDA                182    0.022   4.010   0.635   0.970   0.001   0.030
B I  1JTDA                183    0.184  33.966   0.801   0.970   0.001   0.030
E D  1JTDA                184    0.500  71.992   0.867   0.970   0.001   0.030
B W  1JTDA                185    0.096  23.136   0.522   0.923   0.002   0.076
B M  1JTDA                186    0.064  12.706  -0.671   0.858   0.002   0.139
E E  1JTDA                187    0.415  72.448   0.104   0.782   0.003   0.216
E A  1JTDA                188    0.602  66.384  -0.384   0.522   0.016   0.462
B D  1JTDA                189    0.203  29.195  -1.162   0.181   0.016   0.803
E K  1JTDA                190    0.432  88.862  -0.964   0.052   0.084   0.864
B V  1JTDA                191    0.256  39.316  -1.502   0.052   0.084   0.864
E A  1JTDA                192    0.328  36.190  -0.901   0.184   0.043   0.773
E G  1JTDA                193    0.440  34.628  -0.819   0.257   0.016   0.727
E P  1JTDA                194    0.462  65.544  -0.230   0.339   0.016   0.645
B L  1JTDA                195    0.154  28.161  -0.030   0.455   0.046   0.498
B L  1JTDA                196    0.045   8.203  -0.173   0.455   0.046   0.498
E R  1JTDA                197    0.347  79.463   0.061   0.354   0.048   0.598
E S  1JTDA                198    0.494  57.909  -1.334   0.268   0.043   0.689
B A  1JTDA                199    0.277  30.492  -1.718   0.257   0.016   0.727
B L  1JTDA                200    0.138  25.268  -0.009   0.115   0.016   0.868
B P  1JTDA                201    0.236  33.531  -1.250   0.113   0.043   0.844
E A  1JTDA                202    0.661  72.875  -0.947   0.058   0.017   0.925
E G  1JTDA                203    0.595  46.827  -1.164   0.018   0.019   0.964
B W  1JTDA                204    0.077  18.494   0.401   0.018   0.088   0.893
E F  1JTDA                205    0.253  50.797   0.394   0.021   0.451   0.528
B I  1JTDA                206    0.064  11.859   0.529   0.021   0.756   0.223
B A  1JTDA                207    0.027   2.964  -1.455   0.023   0.655   0.322
B D  1JTDA                208    0.091  13.041  -0.760   0.022   0.552   0.426
B K  1JTDA                209    0.054  11.046  -0.748   0.022   0.359   0.619
B S  1JTDA                210    0.072   8.438  -0.810   0.021   0.279   0.699
B G  1JTDA                211    0.065   5.084  -1.746   0.021   0.279   0.699
B A  1JTDA                212    0.231  25.456  -0.756   0.064   0.216   0.721
B G  1JTDA                213    0.143  11.230  -0.109   0.113   0.087   0.800
E E  1JTDA                214    0.537  93.761  -0.909   0.184   0.043   0.773
E R  1JTDA                215    0.472 108.042  -0.124   0.181   0.016   0.803
B G  1JTDA                216    0.176  13.883  -1.574   0.113   0.043   0.844
B S  1JTDA                217    0.152  17.838  -0.215   0.064   0.216   0.721
B R  1JTDA                218    0.196  44.769   0.280   0.022   0.552   0.426
B G  1JTDA                219    0.028   2.211   0.057   0.003   0.718   0.279
B I  1JTDA                220    0.033   6.123   0.482   0.001   0.900   0.099
B I  1JTDA                221    0.022   4.014   0.368   0.001   0.959   0.040
B A  1JTDA                222    0.017   1.884   0.945   0.001   0.959   0.040
B A  1JTDA                223    0.034   3.747   0.268   0.000   0.983   0.017
B L  1JTDA                224    0.024   4.431   0.185   0.001   0.959   0.040
B G  1JTDA                225    0.261  20.541   1.178   0.001   0.900   0.099
E P  1JTDA                226    0.290  41.165   1.062   0.004   0.420   0.576
E D  1JTDA                227    0.778 112.124  -0.228   0.005   0.015   0.979
E G  1JTDA                228    0.816  64.259  -0.692   0.003   0.003   0.994
E K  1JTDA                229    0.359  73.764   0.475   0.005   0.045   0.951
E P  1JTDA                230    0.263  37.334   0.959   0.004   0.420   0.576
B S  1JTDA                231    0.110  12.915   1.486   0.001   0.900   0.099
B R  1JTDA                232    0.027   6.229   1.199   0.001   0.959   0.040
B I  1JTDA                233    0.019   3.460   1.352   0.001   0.959   0.040
B V  1JTDA                234    0.011   1.768   1.825   0.000   0.983   0.017
B V  1JTDA                235    0.013   1.967   1.544   0.000   0.983   0.017
B I  1JTDA                236    0.017   3.219   1.376   0.001   0.959   0.040
B Y  1JTDA                237    0.060  12.822  -0.005   0.001   0.900   0.099
B T  1JTDA                238    0.054   7.490  -0.272   0.004   0.420   0.576
E T  1JTDA                239    0.457  63.372  -0.234   0.005   0.045   0.951
E G  1JTDA                240    0.486  38.248  -0.487   0.003   0.003   0.994
E S  1JTDA                241    0.486  56.947  -1.481   0.018   0.019   0.964
E Q  1JTDA                242    0.643 114.786  -0.812   0.016   0.005   0.979
E A  1JTDA                243    0.467  51.408  -1.126   0.016   0.005   0.979
E T  1JTDA                244    0.486  67.478   0.414   0.053   0.005   0.942
E M  1JTDA                245    0.328  65.693  -0.021   0.858   0.002   0.139
E D  1JTDA                246    0.668  96.302   1.305   0.970   0.001   0.030
E E  1JTDA                247    0.426  74.422   1.000   0.923   0.002   0.076
B R  1JTDA                248    0.078  17.770  -0.580   0.970   0.001   0.030
E N  1JTDA                249    0.314  45.911   0.394   0.970   0.001   0.030
E R  1JTDA                250    0.532 121.874   2.072   0.970   0.001   0.030
B Q  1JTDA                251    0.100  17.842   1.455   0.970   0.001   0.030
B I  1JTDA                252    0.020   3.793   1.571   0.970   0.001   0.030
E A  1JTDA                253    0.279  30.801   1.509   0.970   0.001   0.030
E E  1JTDA                254    0.483  84.450   1.888   0.970   0.001   0.030
B I  1JTDA                255    0.036   6.660   0.313   0.970   0.001   0.030
B G  1JTDA                256    0.032   2.542   0.009   0.988   0.000   0.012
E A  1JTDA                257    0.389  42.912   1.918   0.988   0.000   0.012
B S  1JTDA                258    0.242  28.398   1.629   0.970   0.001   0.030
B L  1JTDA                259    0.024   4.431   0.536   0.970   0.001   0.030
B I  1JTDA                260    0.155  28.620   0.329   0.923   0.002   0.076
E K  1JTDA                261    0.673 138.477   1.506   0.858   0.002   0.139
E H  1JTDA                262    0.413  75.070   1.064   0.694   0.003   0.303
E W  1JTDA                263    0.352  84.584  -1.525   0.053   0.005   0.942

Explain the output.  Go back.
```

---
